# A Special Section of Reviews From the 2^nd^ Conference on Standards and Ontologies for Functional Genomics (SOFG)

**DOI:** 10.1002/cfg.453

**Published:** 2004-12


					Comparative and Functional Genomics
A Special Section of reviews from
the 2nd
Conference on
Standards and Ontologies for Functional Genomics (SOFG)
Presented by The Jackson Laboratory, University of Pennsylvania and
European Bioinformatics Institute
Edited by: Midori Harris and Helen Parkinson
The goal of the conference was to bring together scientists who are developing standards
and ontologies for describing microarray and other high-throughput functional genomics
experiments. Topics included:
* Ontological Systems: Theory and Development
* Ontologies for Biology Systems
* Ontology Systems Development: Electronic Demonstrations
* Thesauri, Nomenclatures and Biomedical Literature
* Standards and Protocols
* Functional Genomics Applications
* From Resource to Application
Programme Committee
Judith Blake, Ph.D., The Jackson Laboratory
Midori Harris, Ph.D., EMBL, European Bioinformatics Institute
John Macauley, Ph.D., The Jackson Laboratory
Helen Parkinson, Ph.D., EMBL, European Bioinformatics Institute
Susanna-Assunta Sansone, Ph.D., EMBL, European Bioinformatics Institute
Christian Stoeckert, Ph.D., University of Pennsylvania
Abstracts and Presentations are available from:
http://www.sofg.org
The conference was also supported by
National Institute of Environmental Health Sciences-National Center for Toxicology
National Human Genome Research Institute
National Center for Research Resources
Natural Environment Research Council
National Cancer Institute
GlaxoSmithKline
Lab Diet
Test Diet

				

